# An Anti-Human Lutheran Glycoprotein Phage Antibody Inhibits Cell Migration on Laminin-511: Epitope Mapping of the Antibody

**DOI:** 10.1371/journal.pone.0167860

**Published:** 2017-01-06

**Authors:** Yurie Enomoto-Okawa, Yuka Maeda, Nozomi Harashima, Yumika Sugawara, Fumihiko Katagiri, Kentaro Hozumi, Kam Man Hui, Motoyoshi Nomizu, Yuji Ito, Yamato Kikkawa

**Affiliations:** 1 Graduate School of Science and Engineering, Kagoshima University, Kagoshima, Japan; 2 Department of Clinical Biochemistry, Tokyo University of Pharmacy and Life Sciences, Tokyo, Japan; 3 Division of Cellular and Molecular Research, National Cancer Centre, Singapore, Singapore; National Cancer Institute, UNITED STATES

## Abstract

The Lutheran glycoprotein (Lu), also known as basal cell adhesion molecule (B-CAM), is an Ig superfamily (IgSF) transmembrane receptor for laminin α5. Although Lu is not present in normal hepatocytes, its expression is significantly increased in hepatocellular carcinoma (HCC). In this study, we isolated thirteen phage antibodies to Lu from a phage library of peripheral blood from HCC patients, suggesting that these patients produced autoantibodies against endogenous Lu. To characterize the phage antibodies, we determined the Lu domains they recognize. The extracellular domain of Lu contains five IgSF domains, D1-D2-D3-D4-D5. The epitope of one phage antibody (A7) was localized to the D5 domain. The other phage antibodies recognized the D2 domain, which is also recognized by a function blocking mouse monoclonal antibody. One of the antibodies to D2 (C7) inhibited the binding of Lu to ligand, and it also prevented tumor cell migration on laminin-511 (LM-511). However, the C7 scFv purified from the periplasm fraction of bacteria did not exhibit the inhibitory effects, indicating that the scFv form could not sterically inhibit the binding of Lu to LM-511. We also identified the amino acid residues that form the epitope recognized by the C7 phage antibody. Mutagenesis studies showed that Arg^247^ is necessary for forming the epitope. The C7 phage antibody and its epitope may be useful for developing drugs to prevent HCC progression and/or metastasis.

## Introduction

Hepatocellular carcinoma (HCC) is the most common primary tumor of the liver. It is an epithelial cancer originating from hepatocytes. HCC progression results from a multi-step carcinogenic process [1]. Sequential genetic alterations appear to be mainly responsible for HCC progression [2]. Moreover, because HCC progression depends on the interaction between tumor cells and their microenvironment, particularly the surrounding extracellular matrix (ECM) [3], remodeling of the liver microenvironment is a hallmark of HCC pathogenesis. HCC develops in the setting of chronic hepatitis, fibrosis, and cirrhosis, where the hepatic microenvironment is profoundly altered by inflammation and ECM deposition [4]. Several reports have shown that tumor cells, including the HCC cells, are surrounded by ectopic laminins [5, 6]. Laminins are a family of extracellular matrix proteins formed from five α, three β, and three γ chains and are major components of all basal laminae. Although laminin is not present in the normal liver parenchyma, expression of the laminin α5 chain is ectopically observed in well- and poorly-differentiated HCCs [7]. The ectopic deposition of the α5 chain-containing laminins also results in increased levels of its receptors in HCC [7]. Of the receptors for laminin α5, expression of Lutheran glycoprotein/Basal cell adhesion molecule (Lu/B-CAM) is ectopically increased both in well- and poorly-differentiated HCCs, and Lu/B-CAM has served as a candidate HCC specific antigen.

Lu/B-CAM is an Ig superfamily transmembrane protein. Lu has been studied as a blood group antigen and in the context of sickle cell disease [8–12]. B-CAM was also identified as a tumor-associated antigen in ovarian carcinoma [13, 14]. The extracellular domain of Lu/B-CAM contains one variable, one constant-1, and three intermediate Ig-like domains as V-C1-I-I-I [15–17]. Although Lu and B-CAM have the same extracellular and transmembrane domains, B-CAM lacks the last 40 COOH-terminal amino acids of the Lu cytoplasmic tail [13]. Hereafter, because we focus on the extracellular domain shared by Lu and B-CAM, Lu/B-CAM will be referred to as Lu for simplicity. Our recent report showed that Lu and B-CAM promote the migration of lung carcinoma cells on laminin-511 (LM-511), which is composed of α5, β1, and γ1 chains [18]. Of the commercially available antibodies, we also discovered that one monoclonal antibody against Lu can inhibit its binding to laminin α5 [19]. Furthermore, the function-blocking antibody against Lu effectively inhibits the migration of lung carcinoma cells on LM-511. Although the function-blocking antibody derived from mouse hybridoma cells cannot be of clinical use, characterization of the antibody has provided useful information for developing biological drugs to not only inhibit tumor invasion and metastasis, but also inhibit vaso-occlusion in sickle cell disease.

Phage libraries displaying single chain variable fragments (scFv) are powerful tools to screen tumor-associated antigens and other disease antigens. Therefore, phage libraries have also been used for screening scFvs against specific antigens of the HCC cells. However, phage clones specific for the antigens of HCC cells have not been reported yet. The possibility of finding high-affinity phage clones depends on library size, diversity, and source of the immunoglobulin genes. Reasonably, the phage library derived from B cells of tumor patients can provide antibody fragments against specific tumor antigens. Pavoni et al. reported that high-affinity phage clones against tumor antigens are isolated using a library derived from the peripheral blood cells of breast tumor patients [20]. In this study, we attempted to produce a human scFv specific for Lu using phage libraries displaying scFv derived from HCC patients. Several phage clones specific for human Lu were isolated from a phage library of peripheral blood cells. Of these, one phage clone exhibited inhibitory effects on the binding of Lu to its ligand and on LM-511-induced tumor cell migration. We also identified an amino acid residue forming the Lu epitope, recognized by the function-blocking phage clone.

## Materials and Methods

### Antibodies and reagents

Monoclonal antibodies against Lu (mAb87207 and BRIC221) were purchased from R&D systems (Minneapolis, MN) and Serotec (Oxford, UK), respectively. The biotin-labeled anti-filamentous phage M13 fd F1 antibody was purchased from Abcam (Cambridge, UK). Rabbit polyclonal antibody against mouse laminin α5 LEb/L4a domain was a gift from Dr. Jeffrey Miner (Washington University School of Medicine, St Louis, MO). Recombinant LM-511 was prepared as previously described [21]. The recombinant Lu extracellular domain fused with a 6xHis-Tag (Sol-Lu) or an Fc-Tag (Lu-Fc) was produced and characterized as described previously [21, 22].

### Construction of scFv phage libraries

Two types of human scFv-displaying M13 phage libraries were constructed using total RNA from a pool of tumor tissues resected from five patients or from a pool of peripheral blood cells from four HCC patients. Informed consent in writing was obtained from each patient, and the Ethics Committees at the National Cancer Centre, Singapore, approved the experiments. The patients’ ages ranged from 40 to 60 years. Other information, such as gender, Hepatitis virus infection history, and the stage of HCC were not available. Total RNA from tumor tissues and peripheral blood cells was purified with RNAiso Plus (TAKARA, Shiga, Japan). First-strand cDNA was synthesized using SuperScript First-Strand Synthesis System for RT-PCR (ThermoFisher Scientific, Waltham, MA). The V genes for the VH, VLκ, and VLλ were amplified by PCR using appropriate primers based on the human V(D)J gene sequences. The PCR products of the VH and VL gene segments were assembled with a linker DNA that encoded a (Gly_4_Ser)_3_ peptide to construct a single-chain Fv (scFv) gene. Moreover, restriction sites were appended to the assembled scFv genes. The scFv genes were then digested with SfiI and NotI and inserted into the phagemid, pKSTV-EY02, described in our previous study [23]. The phagemid vectors were electroporated into TG1 cells. After the transformation, the diversity of each library was defined. To prepare scFv display phages, the M13KO07 helper phage was added to TG1 cells harboring the constructed phagemids. The phage particles were collected by polyethylene glycol precipitation and then used for biopanning.

### Biopanning and ELISA

96-well ELISA plates (Maxisorp: ThermoFisher Scientific) were coated with 1.0 μg/ml of Sol-Lu and were blocked with 0.5% BSA in phosphate buffered saline (PBS). Both phage libraries (1.0 × 10^10^ pfu) were incubated for 1 h in BSA-coated wells to remove non-specific phages and were then added to Sol-Lu-coated wells. After incubation for 1 h, the wells were washed 5 times with PBS containing 0.1% Tween 20 (PBST). The bound phages were eluted with 0.1 M glycine-HCl (pH 2.0), immediately neutralized with 1 M-Tris HCl (pH 9.1), and then amplified by using them to infect the *Escherichia coli* strain TG1 cells in the log phase of growth. The recovered phages were used for the next round of selection. For ELISA, 96-well ELISA plates were coated with Sol-Lu and blocked with BSA in PBS(-), as described above. The phage solutions were added to the wells and incubated for 1 h. After washing with PBST, the bound phages were detected by addition of biotinylated anti-filamentous phage M13 fd F1 antibody and HRP-conjugated streptavidin, followed by the addition of 0.5 mM 3, 3', 5, 5'-tetramethylbenzidine and 1N HCl. Absorbance was measured at 450 nm using a microplate reader (BIO-RAD, Hercules, CA). Each bar represents the mean of triplicate assays. Error bars indicate standard deviation.

### Purification of soluble scFv

The C7 phage clone was used to infect *E*. *coli* HB2151, and the scFv was expressed by induction with 1 mM IPTG at 37°C. The bacterial cells were collected by centrifugation and disrupted by osmotic shock treatment. The periplasmic fraction was used for affinity purification on His Trap excel (GE Healthcare, Amersham Place, Little Chalfont, Buckinghamshire, UK), according to the manufacturer’s instructions. The purified scFv was dialyzed against PBS(-).

### Surface plasmon resonance analysis

C7 scFv binding was assayed by surface plasmon resonance analysis using a BIAcore T200 system (GE Healthcare). Lu-Fc was immobilized on a CM5 sensor chip (GE Healthcare). Various amounts of C7 scFv in 10 mM HEPES (pH 7.4), containing 150 mM NaCl, 3 mM EDTA, and 0.005% Tween 20, were injected into the Lu-Fc-immobilized flow cell at a flow rate of 50 μl/min. The dissociation constant (K_D_) was determined by a nonlinear fitting method using BIAevaluation software (GE Healthcare).

### Cell culture

A549 and HuH-7 cells were purchased from the Health Science Research Resources Bank (Osaka, Japan). HEK293 cells were obtained from ATCC (Manassas, VA, USA). The cells were maintained in DMEM containing 10% FBS. Two transfectants, Lu/HT1080F and Control/HT1080F cells, were generated previously [18] and maintained in DMEM containing 10% FBS and 100 μg/ml hygromycin (ThermoFisher Scientific).

### Flow cytometry

Cells were removed with cell dissociation buffer (ThermoFisher Scientific) and suspended in PBS(-) containing 0.1% BSA and 1mM EDTA. The suspended cells were incubated with the phage for 1 h at 4°C. After washing with PBS(-) containing 0.1% BSA and 1mM EDTA, the cells were incubated with biotinylated anti-filamentous phage M13 fd F1 antibody for 1 h at 4°C. The bound antibody was detected using Alexa488-labeled streptavidin. The cells were then analyzed on a FACScalibur flow cytometer (Becton Dickinson, San Jose, CA).

### Lu-Fc binding assay

The Lu-Fc binding assay was performed as described previously [19]. Briefly, Lu-Fc was diluted with PBS(-) containing 10 μg/ml of antibody or 5–12 × 10^11^ pfu/ml of phage antibodies and was then placed on adult mouse kidney sections. The α5-containing laminin, the specific ligand of Lu, is enriched in the basement membrane of the kidney. Therefore, we could conveniently perform the binding assay for Lu on the tissue without purifying the α5-containing laminins. Animal studies were approved by the Animal Research Committee of Tokyo University of Pharmacy and Life Sciences (P15-10). Mice were sacrificed under deep anesthesia with Pentobarbital. Bound Lu-Fc was detected with anti-human IgG antibody conjugated to Alexa488 (ThermoFisher Scientific). The anti-laminin α5 polyclonal antibody was used for counter staining. The fluorescence intensities of the bound recombinant proteins were normalized with those of laminin α5 staining in the same areas and quantified using a BZ-analyzer (Keyence, Osaka, Japan).

### Cell migration assays

Cell migration assay using time-lapse video microscopy was performed as described previously [18]. Cells were removed with cell dissociation buffer (ThermoFisher Scientific) and suspended in serum-free DMEM. The cells were then plated on substrata coated with LM-511 (0.8 nM) and blocked with 1% BSA. Two hours post-plating, cell migration was monitored using a Biozero system (Keyence). The nuclear positions were tracked at 10-min intervals for 8 h. Cell velocities were then calculated from the distances of their tracks using Image-J.

### Production of recombinant Lu fused with an Fc-tag and antigen capture ELISA

Full-length cDNAs for human Lu (ThermoFisher Scientific) and mouse Lu (ATCC) were used as templates for PCR. As described in our previous study [19], cDNAs encoding the mutated Lu proteins were generated by PCR using the primer sets listed in S1 Table and were subcloned into the human IgG_1_ Fc expression vector. Transfectants were selected in DMEM containing 10% FBS and 100 μg/ml Zeocin (ThermoFisher Scientific). The recombinant proteins were purified from the serum free conditioned media as described previously [19]. The purified proteins were verified in SDS-PAGE. For antigen capture ELISA, wells were coated with 1 μg/ml of Sol-Lu and blocked with 1% BSA in PBS(-). The diluted monoclonal antibody or scFv was incubated with the recombinant proteins for 1 h at room temperature and then transferred into the Sol-Lu-coated wells. The bound antibody or scFv was detected as described in our previous study [19] or as above.

## Results

### Isolation of Lu-specific scFv display phage clones

**Fig 1:**
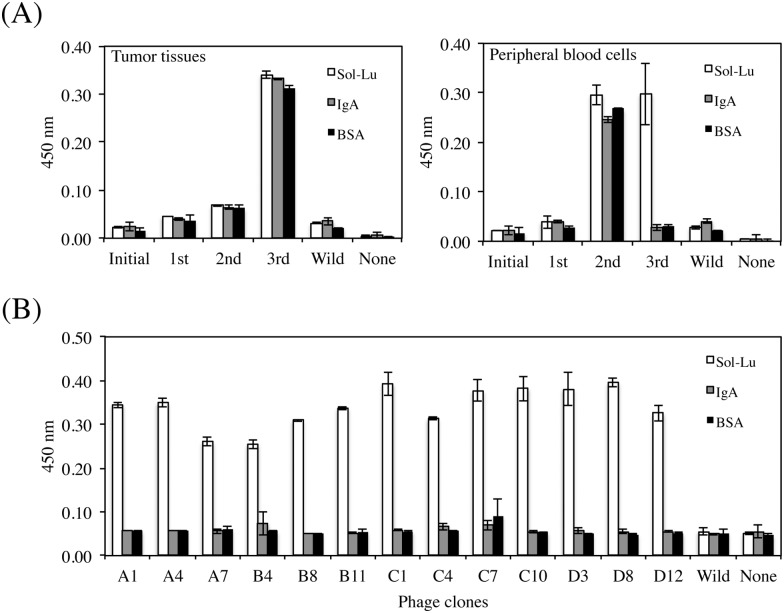
Isolation of human Lu-specific phage clones. (A) Biopanning was performed against Sol-Lu immobilized on wells using HCC patient tissues (left) or peripheral blood cells (right) -derived phage libraries. Enrichment for human Lu-specific phages was only observed from the peripheral blood cell-derived phage library using phage ELISA. (B) Cloning of human Lu-specific phage. After the third round of biopanning, phages were cloned by colony formation. The binding specificities of each phage clone were examined by monoclonal phage ELISA. Thirteen phage clones specific to human Lu were obtained.

**Fig 2:**
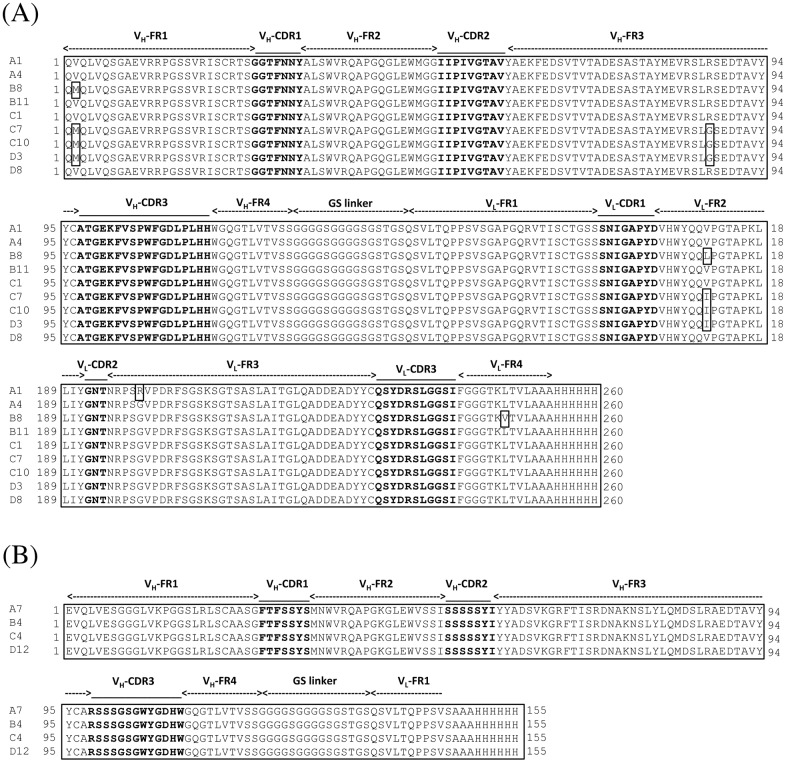
Amino acid sequences of scFvs displayed on human Lu-specific phage clones. (A) Amino acid sequence alignment of scFvs containing the VH and VL domains. The complementary-determining regions (CDR1-CDR3) and the flanking regions (FR1-4) were deduced as described by Kabat et al. [25]. (B) Amino acid sequence alignment of the scFv short forms. The scFv short form lacked the VL domain.

We prepared scFv phage libraries using tumor tissues or peripheral blood cells collected from HCC patients. The sizes of the libraries derived from tumor tissues and peripheral blood cells were 3.6 × 10^8^ and 1.9 × 10^8^, respectively. Three rounds of biopanning against Sol-Lu were performed using each scFv phage library. Sol-Lu-binding phages were enriched in the scFv phage library derived from peripheral blood cells alone (Fig 1). The isolated phage clones were defined using ELISA, and the DNA sequences of the positive clones were determined. Thirteen phage clones were identified that showed specific binding to Sol-Lu but not control proteins (Fig 1). DNA sequence analysis showed that the phage clones were classified into two groups (Fig 2). Although the amino acid sequences of one group contained VH and VL regions, those of the other group lacked the VL region. As shown in a previous study [24], scFv without a VL region may bind to the antigen.

### Epitope mapping of phage antibodies using chimeric mutant Lu proteins

**Fig 3:**
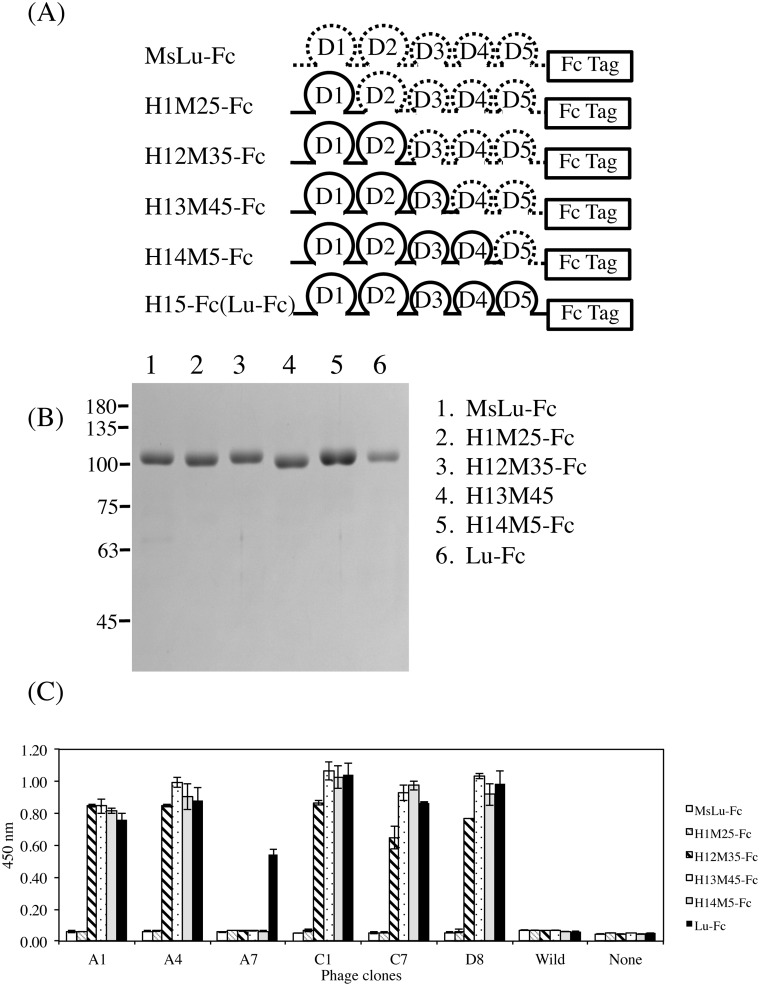
Identification of Lu domains recognized by the anti-Lu phage clones. (A) Diagram of the chimeric mutant proteins designed to narrow the epitopes of the anti-Lu phage clones. For chimeric mutant proteins, the domain(s) of human Lu, Pro^147^-Val^549^, Leu^258^-Val^549^, Leu356-Val^549^, and Leu^442^-Val^549^ was/were replaced with the analogous domain(s) of mouse Lu (MsLu). The chimeric mutant proteins were then fused with an IgG_1_ Fc tag. (B) Purification of a series of recombinant proteins. The purified proteins were subjected to SDS-PAGE on a 7.5% gel. (C) ELISA using the anti-Lu phage clones. The phage clones diluted with PBS(-) were incubated in wells coated with 1 μg/ml of each recombinant protein. The bound phage clones were detected as described in Materials and Methods.

To map the epitopes of isolated phage clones, a series of chimeric mutant proteins was produced in HEK293 cells (Fig 3). The recombinant proteins were tagged with the dimeric Fc form. The isolated phage clones reacted similarly with dimeric Lu as well as with the monomer. Although none of the phage clones reacted with the H1M25-Fc-coated wells, all antibodies except for A7 exhibited immunoactivity for H12M35-Fc (Fig 3). This indicated that the phage antibodies recognized the Lu D2 domain. The clone A7 did not react with chimeric mutant proteins, indicating that it recognized the Lu D5 domain.

### Characterization of anti-Lu D2 domain phage clones

**Fig 4:**
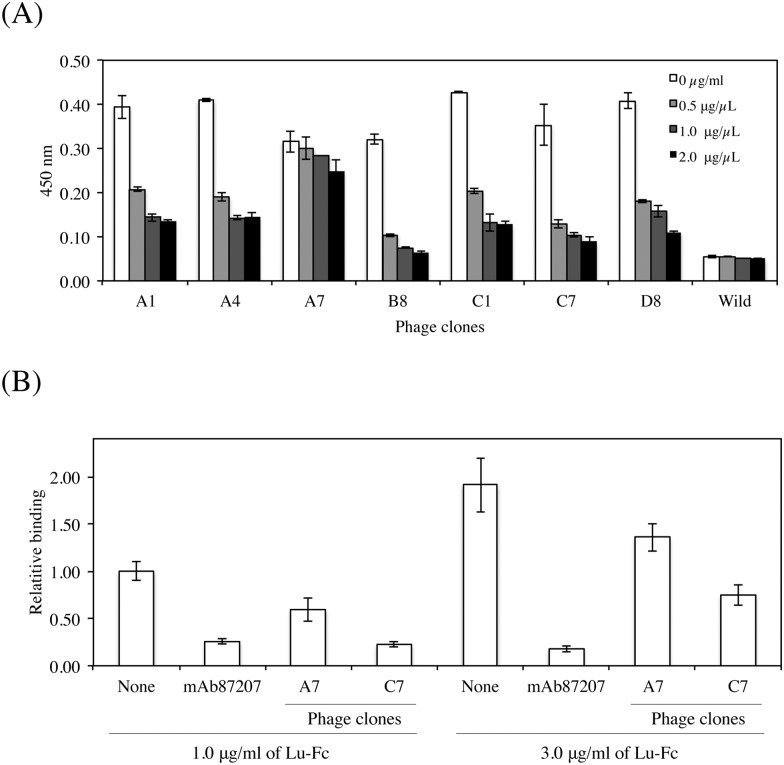
Characterization of anti-Lu phage clones. (A) ELISA using anti-Lu phage clones and mAb87207. Each anti-Lu phage clone was mixed with the diluted antibody at 0.5, 1.0, and 2.0 μg/ml and was added to the wells coated with Sol-Lu. Except for A7, all the phage clones could compete with mAb87207, suggesting that they could inhibit the binding of Lu to laminin α5. (B) Inhibitory effects of the anti-Lu phage clones, A7 and C7, on the binding of Lu-Fc to laminin α5. Binding assays on tissue sections were performed, and the Lu-Fc bound to laminin α5 was quantified as described in Materials and Methods. The binding of Lu-Fc (1 μg/ml) was set to 1.0 in the absence of the anti-Lu phage clones. Each bar represents the mean of triplicate assays. Error bars indicate standard deviation. *, P < 0.05 by *t* test.

We recently reported that a mouse monoclonal antibody (mAb87207) recognizing the Lu D2 domain could inhibit the binding of Lu to laminin α5 [19]. To characterize the isolated phage clones, they were used for ELISA in the presence of mAb87207 (Fig 4). Concentrations of the function-blocking antibody greater than 0.5μg/ml prevented the reactivity of the phage clones, except for the A7 phage clone. This indicates the possibility that the phage clones also have inhibitory effects on the binding of Lu to laminin α5. To examine this possibility, binding assays on tissue sections were performed in the presence of the phage clones (Fig 4). Although the A7 phage clone recognized the Lu D5 domain, it slightly inhibited the binding of Lu-Fc to laminin α5. The phage clone C7, significantly inhibited Lu binding similar to mAb87207.

### Inhibitory effect of the C7 phage clone on tumor cell migration on LM-511

**Fig 5:**
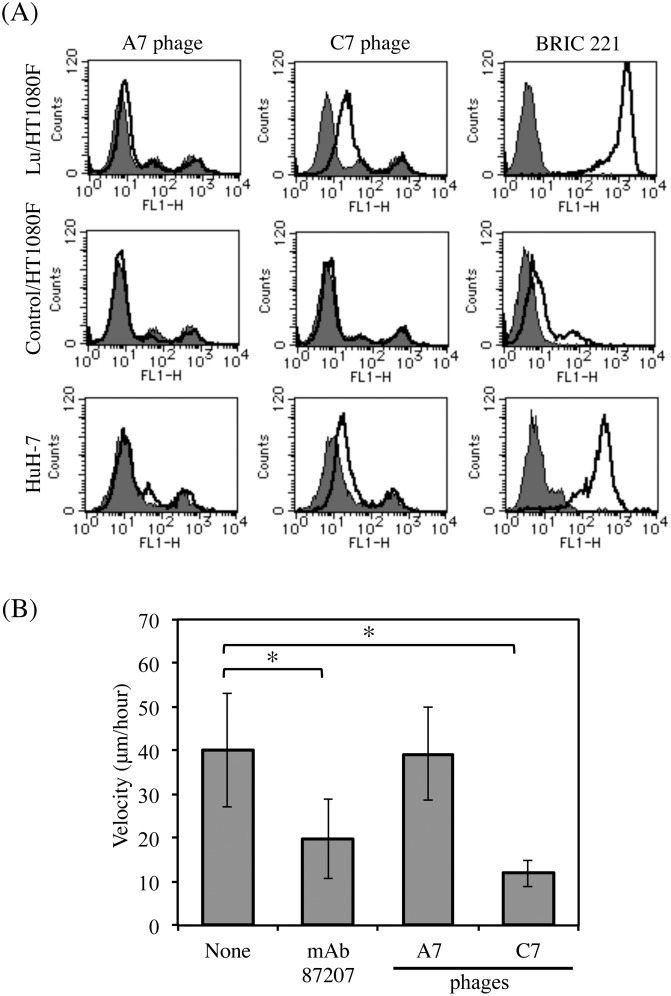
Effects of anti-Lu phage antibodies on tumor cell migration. (A) Flow cytometric analyses using anti-Lu phage antibodies. The transfectants expressing Lu and HuH-7 cells were incubated with the indicated primary antibodies (A7, 2.0 × 10^10^ pfu/2.0 × 10^5^ cells; C7, 2.0 × 10^10^ pfu/2.0 × 10^5^ cells; BRIC221, 10μg/2.0 × 10^5^ cells) and then with Alexa 488-conjugated secondary antibody for flow cytometric analysis. The expression of Lu is shown as a solid line. The gray area indicates the negative control. (B) Migration of A549 cells on LM-511 in the presence of either mAb87207 or the anti-Lu phage antibodies. After the cells adhered to substrata coated with LM-511 (0.8 nM), antibodies were added to the media (A7, 1.2 × 10^11^ pfu/ml; C7, 5 × 10^10^ pfu/ml; mAb87207, 10 μg/ml). Cell movements were monitored by time-lapse video microscopy and were quantified as described in Materials and Methods. Ten cells were randomly selected in each field. *, P < 0.01 by *t* test.

To examine whether the phage clones could react with Lu expressed on the cell surface, we used transfectants expressing Lu and a HCC cell line (Fig 5A). Flow cytometric analysis showed that although the phage clone C7 recognized the transfectants expressing Lu, the phage clone A7 could not bind to Lu. Because the size of the filamentous phage is about 800 nm, the phage clone A7 that recognizes the Lu D5 domain, may not be able to access the juxtamembrane region of Lu. The phage clone C7, significantly bound to the HuH-7 cells expressing Lu. We also examined the effects of the phage clone C7 on LM-511-induced tumor cell migration (Fig 5B). We performed the cell migration assay using the HCC cells, HuH-7. However, the velocity of HCC cell migration was not sufficient to examine the inhibitory effects of the antibodies or the phage clones in the cell migration assay. Therefore, as described in our previous study [26], we used the A549 cells for the cell migration assay using the phage clone C7. The tracks of the lung adenocarcinoma cells, A549, on LM-511 were traced using time-lapse video microscopy. The phage clone C7 inhibited tumor cell migration on LM-511 more effectively than did mAb87207. Because the phage clone A7 did not react with Lu expressed on the cell surface, it was used as a control.

### Isolation and characterization of C7 scFv

**Fig 6:**
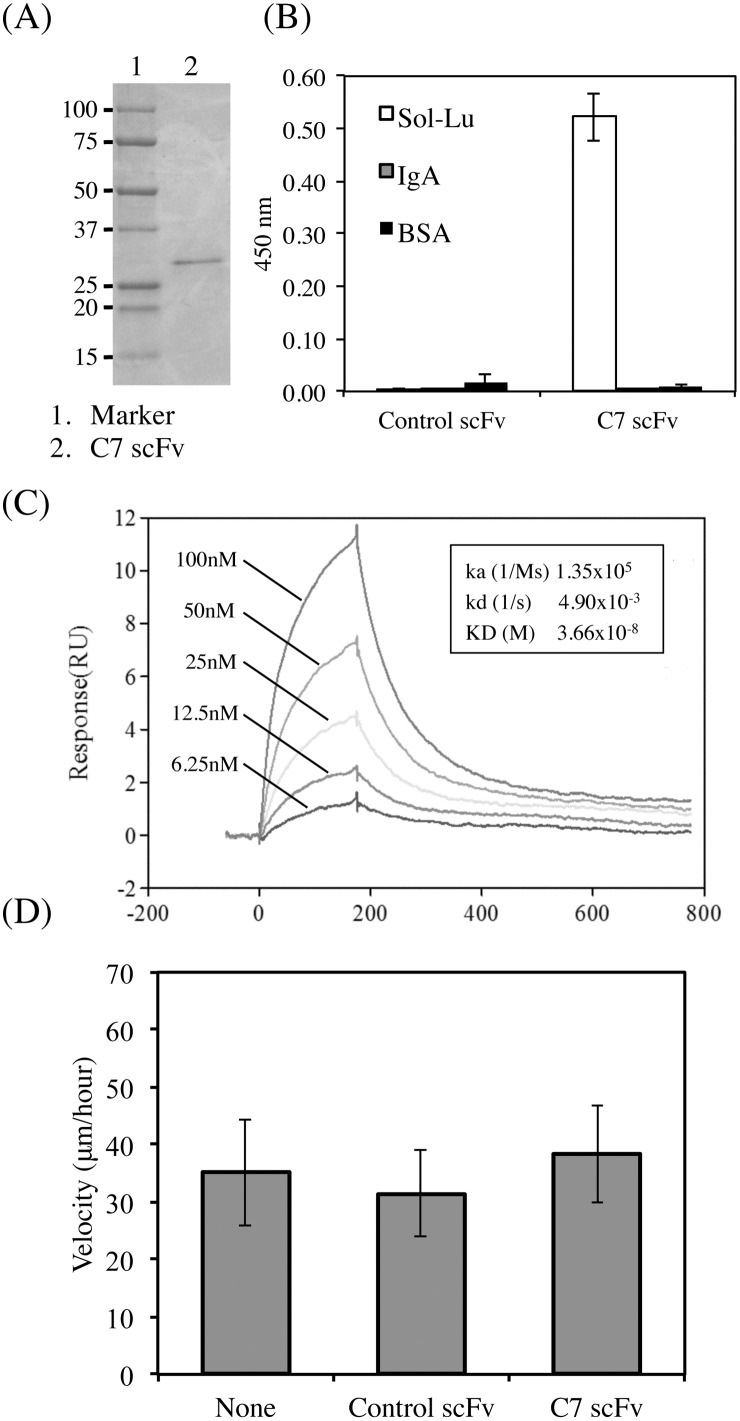
Characterization of C7 scFv purified from the *E*. *coli* extract. (A) Purification of C7 scFv from *E*. *coli* extracts. The purified protein was subjected to SDS-PAGE on a 10–20% gradient gel under reducing conditions and was stained with Coomassie brilliant blue. (B) ELISA using the purified C7 scFv. C7 scFv diluted at 1 μg/ml was incubated in wells coated with 1 μg/ml of Lu-Fc. (C) SPR analysis of C7 scFv. The sensorgrams represent the association and dissociation upon injecting different concentrations of C7scFv (6.25, 12.5, 25, 50, and 100 nM) to immobilized Lu-Fc. The rate constants, Ka and Kd, and KD are shown in the sensorgram. (D) Migration of A549 cells on LM-511 in the presence of C7 scFv. After the cells adhered to substrata coated with LM-511 (0.8 nM), control or C7 scFv was added to the media (30 μg/ml). Cell movements were evaluated as described above.

To purify the clone C7 scFv, the non-suppressor host, *E*. *coli* HB2151 cells infected with the phage clone were cultivated in a shaker flask. Protein expression was induced by IPTG. The soluble scFv protein was purified from the periplasmic extracts of *E*. *coli* as described in Materials and Methods. SDS-PAGE analysis of the purified C7 scFv revealed a single protein band corresponding to the expected molecular size of 29–30 kDa (Fig 6). C7 scFv was verified as monomer using size exclusion chromatography (data not shown). The binding activity of the purified protein was confirmed using ELISA (Fig 6). The purified C7 scFv was found to bind Lu-Fc. We also measured the binding affinity of C7 scFv using surface plasmon resonance (SPR) (Fig 6). Lu-Fc was coupled to the Biacore CM5 sensor chip, and various concentrations of C7 scFv were run over the chip. The Ka and Kd were 1.35 × 10^5^ s^-1^M^-1^ and 4.9 × 10^−3^ s^-1^, respectively. The value of KD is 3.66 × 10^−8^ M. Furthermore, we examined the effects of C7 scFv on LM-511-induced tumor cell migration (Fig 6). Although the phage form could inhibit tumor cell migration, C7 scFv did not possess any inhibitory effects. Because the molecular size of scFv was smaller than that of whole IgG, it may not prevent the binding of Lu spatially.

### Fine mapping of the C7 scFv epitope on the Lu D2 domain

**Fig 7:**
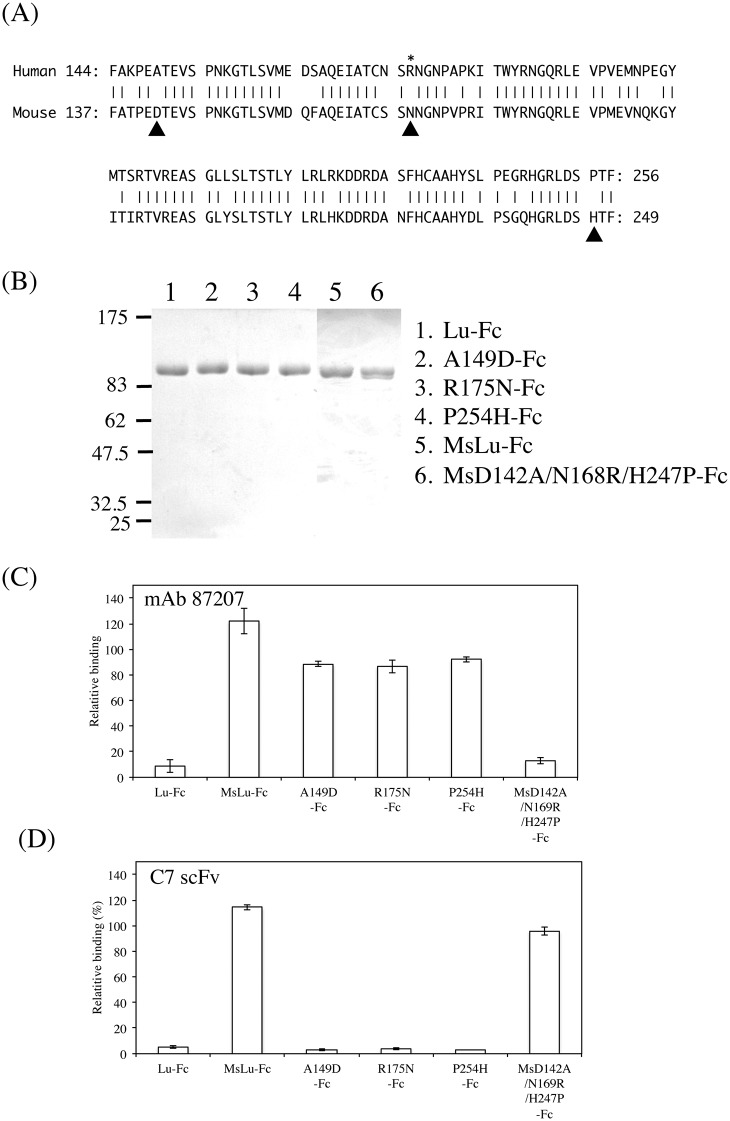
The binding of C7 scFv to the epitope of function-blocking antibody against Lu. (A) Alignment of the human and mouse Lu D2 domain sequences. Amino acids that are identical across species are connected with vertical lines. Arrowheads represent the amino acid residues forming the epitope of mAb87207. For A149D-Fc, R175N-Fc, and P254H-Fc, Ala^149^, Arg^175^, and Pro^254^ in Lu-Fc were substituted with the corresponding mouse residues, Asp, Asn, and His, respectively. For MsD142A/N168R/H247P-Fc, Asp^142^, Asn^168^, and His^247^ in MsLu-Fc were substituted with the corresponding human residues Ala, Arg, and Pro, respectively. (B) SDS-PAGE analysis of the mutant proteins purified from the conditioned media of the HEK293 transfectants. (C) ELISA using mAb87207 mixed with the mutant Lu-Fc. The mutant proteins were mixed with the antibody diluted at 1 μg/ml and were added to the wells coated with 3 μg/ml of Sol-Lu. MsD142A/N168R/H247P-Fc restored the binding of mAb87207 to the antigen. (D) ELISA using C7 scFv mixed with the mutant Lu-Fc. The mutant proteins were mixed with the diluted C7 scFv at concentration of 0.6 μg/ml. C7 scFv did not bind to the epitope of mAb87207.

**Fig 8:**
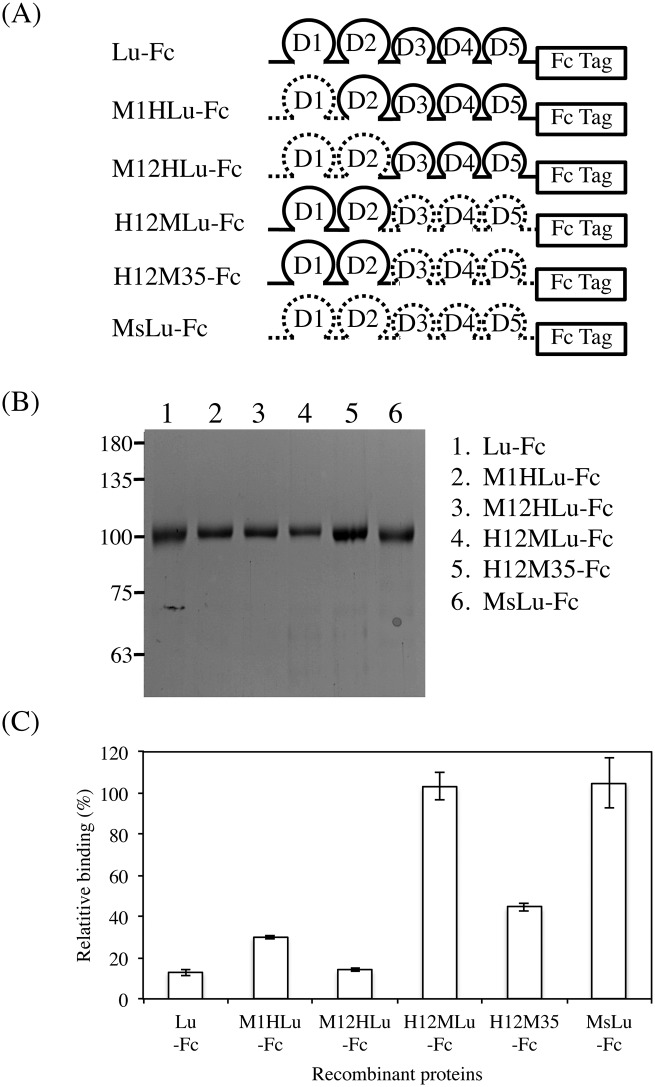
Mapping of the region recognized by C7 scFv. (A) Diagram of the chimeric mutant proteins designed to narrow the epitopes of C7 scFv. For M1HLu-Fc, M12HLu-Fc, H12MLu-Fc, and H12M35-Fc, the domain(s) of human Lu, Glu^32^-Cys^172^, Glu^32^-Cys^237^, Cys^237^-Val^549^, and Leu^258^-Val^549^ was/were replaced with the analogous domain(s) of mouse Lu. The chimeric mutant proteins were fused with an IgG_1_ Fc tag. (B) SDS-PAGE analysis of the chimeric mutant proteins purified from conditioned media. (C) ELISA using C7 scFv mixed with various recombinant proteins. The chimeric mutant proteins were mixed with the diluted C7 scFv at a concentration of 0.6 μg/ml and were added to wells coated with Lu-Fc. The epitope of C7 scFv was localized to the region from Ala^238^ through His^257^.

**Fig 9:**
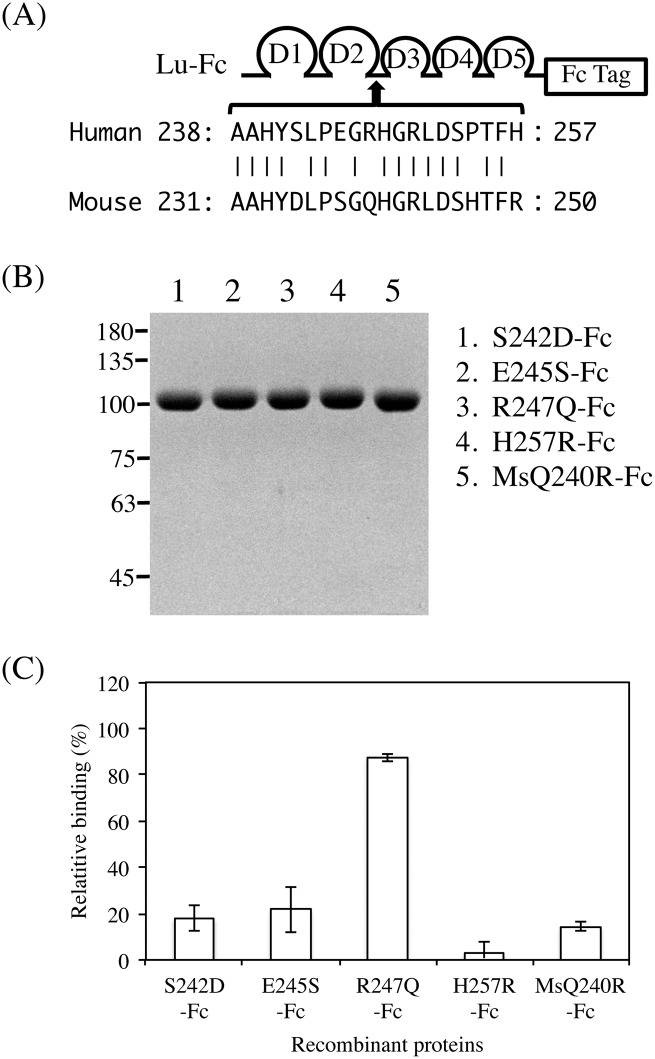
Fine mapping of the epitope recognized by C7 scFv. (A) Alignment of the human and mouse Lu amino acid sequences. Amino acids that are identical across species are connected with vertical lines. Ser^242^, Glu^245^, Arg^247^, and His^257^ in Lu-Fc were substituted with the corresponding mouse residues, Asp, Ser, Gln, and Arg, respectively. Gln^240^ in MsLu-Fc was substituted with Arg, the corresponding human residue. (B) Purification of the mutant proteins. The mutant proteins purified from conditioned media were subjected to SDS-PAGE on a 7.5% gel. (C) ELISA using C7 scFv mixed with various recombinant proteins. The mutant proteins were mixed with the diluted C7 scFv and were added to the wells coated with Lu-Fc. C7 scFv did not react with R247Q-Fc, indicating that Arg^247^ is required for immunoreactivity.

Our previous study showed that Arg^175^, the site of LU4/LU-4, was a pivotal amino acid in the epitope recognized by the function-blocking antibody, mAb87207 [19]. In addition, we recently identified two amino acid residues, Ala^149^ and Pro^254^, forming the epitope of mAb87207 (Fig 7). We also produced a recombinant protein, MsD142A/N168R/H247P-Fc, in which Asp^142^, Asn^168^, and His^247^ in mouse Lu-Fc were substituted with Ala, Arg, and Pro, the corresponding human residues. The recombinant protein restored the immunoreactivity for mAb87207, indicating that Ala^149^, Arg^175^, and Pro^245^ formed the epitope of the antibody. Because C7 scFv competitively bound to Lu in the presence of mAb87207, we considered the possibility that these amino acid residues could be part of the C7 scFv epitope (Fig 7). However, C7 scFv did not react with the epitope of mAb87207. To narrow down the region on the Lu D2 domain recognized by C7 scFv, we produced a series of chimeric recombinant Lu fragments with portions of the human Lu D2 domain replaced with analogous portions of mouse Lu. ELISA using the chimeric proteins showed that the epitope of C7 scFv was localized in the region from Ala^238^ through His^257^ (Fig 8). As shown above, C7 scFv did not react with mouse Lu. Therefore, the differences between the human and mouse Lu amino acid sequences (Fig 9) might be responsible for its immunogenicity. Ser^242^, Glu^245^, Arg^247^, Pro^254^, and His^257^ differ from the corresponding mouse Lu residues. We produced a series of mutated human Lu-Fc proteins substituted with the corresponding amino acids found in mouse Lu (Fig 9). ELISA using the recombinant proteins showed that Arg^247^ was essential for forming the epitope of C7 scFv. We also produced a recombinant protein, MsQ240R-Fc, in which Gln^240^ in mouse Lu-Fc was substituted with Arg, the corresponding human residue. The recombinant protein restored the immunoreactivity for C7 scFv, indicating that Arg^247^ was essential to form the epitope of scFv.

## Discussion

To identify HCC-associated antigens at the cell surface, we constructed human scFv phage display libraries using tumor tissues and peripheral blood cells from HCC patients. Although biopanning was performed using HCC cell lines, enrichment for phage clones specific to HCC cells was not observed (unpublished data). After cell-based biopanning, as per our strategy we targeted cell surface molecules that are strongly expressed on HCC cells. We have previously reported that the expression of Lu is increased in HCC [7]. In addition, this cell surface molecule is observed in poorly- differentiated through well-differentiated HCCs. Therefore, of several candidates, Lu was chosen as an antigen for the selection of phage clones. After biopanning using a recombinant Lu protein, several clones binding to Lu were isolated from the peripheral blood cell-derived phage library, but no positive phage clones were obtained from the tumor tissue-derived library. The immune system often produces antibodies against mutated and aberrantly expressed proteins in tumor tissues. These results suggest that B cells produce autoantibodies against Lu in HCC patients. In future, we will examine whether such autoantibodies exist in the sera of HCC patients. Pavoni et al. showed that breast tumor tissues containing B cells are an efficient source for constructing phage display libraries [20]. Based on their results, we constructed a library derived from HCC patient tumor tissues. However, our results suggest that B cells do not infiltrate HCC tumor tissues.

The isolated phage clones were classified into two groups. In one group, scFv consisted of the VH and VL regions, and recognized the Lu D2 domain. In the other group, scFv lacking the VL region recognized the Lu D5 domain. In a previous study we showed that although mAb87207 recognizes the Lu D2 domain, which does not harbor the major binding sites for laminin α5, it still inhibits the binding of Lu to LM-511 spatially [19]. As anticipated, the phage clone C7 recognizing the Lu D2 domain exhibited inhibitory effects on the binding of Lu to laminin α5. However, despite its binding to Lu, the C7 scFv lost these inhibitory effects. Similar to mAb87207, the inhibitory mechanism of the phage clone C7 also seems to involve steric hindrance of Lu binding to laminin α5. We also tried to increase the molecular size of C7 scFv with a 6xHis tag antibody. However, the complex did not reveal any inhibitory effects on cell migration. These results suggest that not only the molecular size but also the conformation are critical for successful inhibition by molecules recognizing the Lu D2 domain. To impart the ability to inhibit Lu binding to LM-511, C7 scFv might need to be incorporated into full antibody or macromolecules. A patch of negatively charged residues at the base of the D2 domain and the top of the D3 domain is required for the laminin α5 binding [16]. Here, the phage clone A7 recognizing the Lu D5 domain also inhibited the binding of Lu to laminin α5. Zen et al. have previously reported that the fifth IgSF (D5) domain is required for binding [12]. Although the binding site for laminin α5 seems to be formed by the D2 and D3 domains, it is possible that two distinct binding sites of Lu could interact with laminin α5. Our results suggest that the B cells of HCC patients produce function-blocking autoantibodies against Lu.

**Fig 10:**
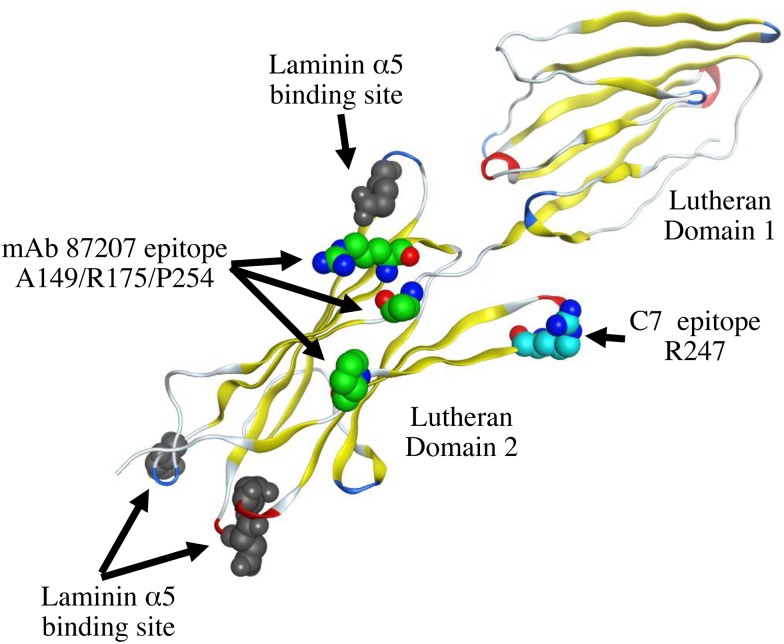
Mapping the amino acid residue recognized by C7 scFv on a three-dimensional model of Lu D1 and D2 domains. An amino acid residue of the C7 scFv epitope mapped on the crystal structure of Lu D1 and D2 domains (Protein Data Bank code 2PET) [16]. The location of Arg^247^ is not very close to the epitope of the mAb87207 epitope. The binding sites of laminin α5 on Lu D2 domain are located on the opposite side of Arg^247^.

Nineteen antigens numbered from LU1 to LU21, with two of these declared obsolete, have been identified as the antigens of the Lutheran blood group system [27]. Our results suggest that HCC patients produce autoantibodies to Lu. Therefore, we suspected that the amino acids relevant to these determinants would be particularly immunogenic. However, although C7 scFv recognized the Lu D2 domain that contains the epitopes for the human alloantibodies LU4, LU8, LU14, and LU16, they did not represent the epitope of C7 scFv (data not shown). Our mutagenesis studies showed that Arg^247^ was a pivotal amino acid in the epitope recognized by C7 scFv. Arg^247^ may thus be a previously unrecognized antigen of the Lutheran blood group system. Mapping of the C7 scFv epitope on the crystal structure of the Lu D1 and D2 domains show that it is located in a β-sheet (Fig 10). The epitope recognized by a typical monoclonal antibody is usually formed by several residues. To narrow the region of the epitope, we synthesized a peptide corresponding to the amino acid sequence from Ala^238^ through His^257^. However the peptide was not recognized by the C7 phage clone, indicating that the epitope sterically consisted of several amino acids, including Arg^247^ (data not shown). We used the differences between the human and mouse Lu amino acid sequences to identify the epitope of C7 scFv. However there are no longer candidate amino acids that we can choose. In future experiments, Ala or Gly substitution in the amino acid sequence around Arg^247^ will be required for the complete epitope mapping.

The scFv could be useful for the design and construction of anti-cancer antibodies, immunotoxins, and drug delivery systems for therapeutic purposes. C7 scFv would be especially useful for the design of an antibody drug to suppress HCC progression. The scFv form is less stable in vivo than the full antibody form. In the future, we will have to produce a C7 scFv-incorporated full antibody to determine its efficacy in vivo. Lu has been studied as an antigen of the Lutheran blood group system as well as in the context of sickle cell disease [27]. Vaso-occlusion in patients has been seemed to be involved in the increased adhesion of sickled red cells to laminin α5 via Lu binding [28, 29]. Therefore, the generation of inhibitory antibodies or drugs is required for the management of vaso-occlusion in sickle cell disease. C7 scFv and its epitope should be useful for developing engineered antibodies and small molecules to inhibit the vaso-occlusion commonly observed in sickle cell disease.

## Conclusion

The development of targeted molecular therapies for cancer treatment requires the identification of specific tumor antigens. Our approach using scFv phage antibodies revealed that Lu is a candidate-tumor-specific antigen for HCC. Although C7 scFv specific for Lu did not influence its cellular interactions with LM-511, the phage antibody displaying the C7 scFv prevented tumor cell migration on LM-511. C7 scFv and its epitope will be useful for the construction of function-blocking molecules and the design of drug delivery systems for treating HCC.

## Supporting Information

S1 Table Primer sets. (DOCX)
