# Supplementary material for: An Anti-Human Lutheran Glycoprotein Phage Antibody Inhibits Cell Migration on Laminin-511: Epitope Mapping of the Antibody
Source: PLoS One. 2017 Jan 6;12(1):e0167860. doi: 10.1371/journal.pone.0167860 (PMC5218393; doi:10.1371/journal.pone.0167860)
Supplement: S1 Table — (DOCX) [file pone.0167860.s001.docx]

| Recombinant proteins | Primer | Sequence (5'-3') |
| --- | --- | --- |
| MsLu-Fc | MLU01 | CGCAATTGGCCACCATGGAACCCCCTGACGCCCGCGCA |
|  | MLU02 | CGTCTAGACCCACTCCAGCCTGGGCAGTCTGAGG |
| H1M25-Fc | HLU101 | CGCAATTGGCCACCATGGAGCCCCCGGACGCACCG |
|  | HLU138 | AGACACCTCCGTATCCTCTGGCTTTGCAAACACGTTGAGCCG |
|  | MLU21 | CGCCTCAACGTGTTTGCAAAGCCAGAGGATACGGAGGTGTCT |
|  | MLU02 | CGTCTAGACCCACTCCAGCCTGGGCAGTCTGAGG |
| H12M35-Fc | HLU101 | CGCAATTGGCCACCATGGAGCCCCCGGACGCACCG |
|  | HLU140 | CGTGGGATAGTGCAGGGTGAGGTGGAAGGTGGGGCTGTCCAG |
|  | MLU23 | CTGGACAGCCCCACCTTCCACCTCACCCTGCACTATCCCACG |
|  | MLU02 | CGTCTAGACCCACTCCAGCCTGGGCAGTCTGAGG |
| H13M45-Fc | HLU101 | CGCAATTGGCCACCATGGAGCCCCCGGACGCACCG |
|  | HLU142 | ATCCAGGTAGGCCACGTGCAGCTCCAGCGTCTTGGAGAGCTG |
|  | MLU25 | CAGCTCTCCAAGACGCTGGAGCTGCACGTGGCCTACCTGGAT |
|  | MLU02 | CGTCTAGACCCACTCCAGCCTGGGCAGTCTGAGG |
| H14M5-Fc | HLU101 | CGCAATTGGCCACCATGGAGCCCCCGGACGCACCG |
|  | HLU144 | TGGTGCTCCTTGGACGATGAGCGTGAAGTTCTGGGTGCGGCT |
|  | MLU27 | AGCCGCACCCAGAACTTCACGCTCATCGTCCAAGGAGCACCA |
|  | MLU02 | CGTCTAGACCCACTCCAGCCTGGGCAGTCTGAGG |
| H15-Fc(Lu-Fc) | HLU03 | GGAATTCGCCACCATGGAGCCCCCGGACGCACCG |
|  | HLU34 | CGTCTAGACCCACTCCAGCCTGGGAGGTCTG |
| A149D-Fc | HLU03 | GGAATTCGCCACCATGGAGCCCCCGGACGCACCG |
|  | HLU108 | TTTGTTGGGGGAGACCTCAGTATCCTCTGGCTTTGCAAACACGTT |
|  | HLU79 | AACGTGTTTGCAAAGCCAGAGGATACTGAGGTCTCCCCCAACAAA |
|  | HLU34 | CGTCTAGACCCACTCCAGCCTGGGAGGTCTG |
| R175N-Fc | HLU03 | GGAATTCGCCACCATGGAGCCCCCGGACGCACCG |
|  | HLU96 | GTTCCCGTTGTTGCTGTTGCAGGTGGCGATCTC |
|  | HLU55 | TGCAACAGCAACAACGGGAACCCGGCCCCCAAG |
|  | HLU34 | CGTCTAGACCCACTCCAGCCTGGGAGGTCTG |
| P254H-Fc | HLU03 | GGAATTCGCCACCATGGAGCCCCCGGACGCACCG |
|  | HLU110 | GTGCAGGGTGAGGTGGAAGGTATGGCTGTCCAGGCGGCCGTGGCG |
|  | HLU81 | CGCCACGGCCGCCTGGACAGCCATACCTTCCACCTCACCCTGCAC |
|  | HLU34 | CGTCTAGACCCACTCCAGCCTGGGAGGTCTG |
| MsD142A/N169R/H247P-Fc | MLU01 | CGCAATTGGCCACCATGGAACCCCCTGACGCCCGCGCA |
|  | MLU16 | TTTGTTGGGAGACACCTCCGTGGCCTCTGGTGTTGCAAACACACG |
|  | MLU13 | CGTGTGTTTGCAACACCAGAGGCCACGGAGGTGTCTCCCAACAAA |
|  | MLU12 | CCGAGGCACGGGGTTTCCGTTCCGGCTGCTGCAGGTGGCAATCTC |
|  | MLU09 | GAGATTGCCACCTGCAGCAGCCGGAACGGAAACCCCGTGCCTCGG |
|  | MLU18 | GTGCAGGGTGAGGCGAAAGGTGGGGCTGTCCAGGCGGCCATGTTG |
|  | MLU15 | CAACATGGCCGCCTGGACAGCCCCACCTTTCGCCTCACCCTGCAC |
|  | MLU02 | CGTCTAGACCCACTCCAGCCTGGGCAGTCTGAGG |
| M1HLu-Fc | MLU01 | CGCAATTGGCCACCATGGAACCCCCTGACGCCCGCGCA |
|  | MLU06 | GTTCCCGTTCCGGCTGTTGCAGGTGGCAATCTCCTGGGC |
|  | HLU59 | GCCCAGGAGATTGCCACCTGCAACAGCCGGAACGGGAAC |
|  | HLU34 | CGTCTAGACCCACTCCAGCCTGGGAGGTCTG |
| M12HLu-Fc | MLU01 | CGCAATTGGCCACCATGGAACCCCCTGACGCCCGCGCA |
|  | MLU20 | GGGCAGGCTGTAGTGGGCGGCACAGTGGAAGTTGGCATCCCGATCATC |
|  | HLU113 | GATGATCGGGATGCCAACTTCCACTGTGCCGCCCACTACAGCCTGCCC |
|  | HLU34 | CGTCTAGACCCACTCCAGCCTGGGAGGTCTG |
| H12MLu-Fc | HLU101 | CGCAATTGGCCACCATGGAGCCCCCGGACGCACCG |
|  | HLU104 | CAAGTCGTAGTGAGCAGCACAGTGGAAGCTGGCGTCTCGGTC |
|  | HLU73 | TGCAACAGCGCGAACGGGAACCCGGCCCCCAAG |
|  | MLU02 | CGTCTAGACCCACTCCAGCCTGGGCAGTCTGAGG |
| S242D-Fc | HLU03 | GGAATTCGCCACCATGGAGCCCCCGGACGCACCG |
|  | HLU146 | GCCGTGGCGGCCCTCGGGCAGGTCGTAGTGGGCGGCGCAGTGGAA |
|  | HLU107 | TTCCACTGCGCCGCCCACTACGACCTGCCCGAGGGCCGCCACGGC |
|  | HLU34 | CGTCTAGACCCACTCCAGCCTGGGAGGTCTG |
| E245S-Fc | HLU03 | GGAATTCGCCACCATGGAGCCCCCGGACGCACCG |
|  | HLU148 | GTCCAGGCGGCCGTGGCGGCCTGAGGGCAGGCTGTAGTGGGCGGC |
|  | HLU109 | GCCGCCCACTACAGCCTGCCCTCAGGCCGCCACGGCCGCCTGGAC |
|  | HLU34 | CGTCTAGACCCACTCCAGCCTGGGAGGTCTG |
| H257R-Fc | HLU03 | GGAATTCGCCACCATGGAGCCCCCGGACGCACCG |
|  | HLU136 | CGTGGGATAGTGCAGGGTGAGGCGGAAGGTGGGGCTGTCCAGGCG |
|  | HLU105 | CGCCTGGACAGCCCCACCTTCCGCCTCACCCTGCACTATCCCACG |
|  | HLU34 | CGTCTAGACCCACTCCAGCCTGGGAGGTCTG |
| R247Q-Fc | HLU03 | GGAATTCGCCACCATGGAGCCCCCGGACGCACCG |
|  | HLU150 | GGGGCTGTCCAGGCGGCCGTGTTGGCCCTCGGGCAGGCTGTAGTG |
|  | HLU111 | CACTACAGCCTGCCCGAGGGCCAACACGGCCGCCTGGACAGCCCC |
|  | HLU34 | CGTCTAGACCCACTCCAGCCTGGGAGGTCTG |
| MsQ240R-Fc | MLU01 | CGCAATTGGCCACCATGGAACCCCCTGACGCCCGCGCA |
|  | MLU26 | ATGGCTGTCCAGGCGGCCATGGCGGCCTGAGGGCAAGTCGTAGTG |
|  | MLU33 | CACTACGACTTGCCCTCAGGCCGCCATGGCCGCCTGGACAGCCAT |
|  | MLU02 | CGTCTAGACCCACTCCAGCCTGGGCAGTCTGAGG |
